# A Peristaltic Micro Pump Driven by a Rotating Motor with Magnetically Attracted Steel Balls

**DOI:** 10.3390/s90402611

**Published:** 2009-04-15

**Authors:** Min Du, Xiongying Ye, Kang Wu, Zhaoying Zhou

**Affiliations:** State Key Laboratory of Precision Measurement Technology and Instruments, Department of Precision Instruments and Mechanology, Tsinghua University, Beijing 100084, P.R. China; E-Mails: dum07@mails.tsinghua.edu.cn (M.D.); wuk05@mails.tsinghua.edu.cn (K.W.); zhouzy@mail.tsinghua.edu.cn (Z.Y.Z.)

**Keywords:** Micropump, microfluidics, PDMS, lab-on-a-chip, soft lithography

## Abstract

In this paper, we present a membrane peristaltic micro pump driven by a rotating motor with magnetically attracted steel balls for lab-on-a-chip applications. The fabrication process is based on standard soft lithography technology and bonding of a PDMS layer with a PMMA substrate. A linear flow rate range ∼490 μL/min was obtained by simply varying the rotation speed of a DC motor, and a maximum back pressure of 592 Pa was achieved at a rotation speed of 43 rpm. The flow rate of the pump can also be adjusted by using steel balls with different diameters or changing the number of balls. Nevertheless, the micro pump can also work in high speed mode. A high back pressure up to 10 kPa was achieved at 500 rpm using a high speed DC motor, and an utmost flow rate up to 5 mL/min was reached.

## 1. Introduction

Micro pumps, an important actuating device in microfluidics, play a key role in lab-on-a-chip and μTAS. Over the past decades, various micro pump designs have been developed [1–3], mostly mechanical displacement pumps and dynamic pumps. Mechanical displacement pumps usually consist of two valves and an actuation chamber and are driven by pneumatic [4], electromagnetic [5], piezoelectric [6], electrostatic actuation [7] and so on [8]. These pumps often have the problem of a large dead volume. Dynamic pumps can be classified as electroosmotic [9], centrifugal [10], electrohydrodynamic [11], ultrasonic [12], magnetohydrodynamic [13] pumps and so on. Dynamic pumps have the advantages of relatively simple design, no mechanical moving parts and a smaller dead volume, but many require a high voltage and/or a charged liquid. Peristaltic micro pumps have been demonstrated as another group of mechanical displacement pumps [14–22], which can be generally divided into two categories: discontinuous peristaltic pumps driven by controlled compression of several cooperative chambers [14–19], and continuous peristaltic pumps driven by compression of a single continuous channel [20–22]. Kim *et al*. reported a continuous peristaltic micro pump using magnetic fluids (MF) [20]. With this MF micropump it is hard to achieve high back pressures. Koch *et al*. presented another kind of continuous peristaltic micro pumps based on direct compression of a tube or a PDMS channel using rollers connected with a motor [21]. However, roller-driven micro pumps need extra lubrication.

Yobas *et al*. demonstrated a simple planar peristaltic pump fabricated with PDMS via soft lithography and driven by permanent magnets [22]. It achieved a flow rate of up to about 1,000 μL/min, with good overall performance in a low flow rate range. However, the relatively large chip size (as large as a CD) may limit its applications.

Here we report another design of an improved PDMS peristaltic micro pump with magnetically attracted steel balls driven by permanent magnets. Our main goal is to minimize the chip size and still keep a wide flow rate range. The pump has a wide adjustable flow rate range using different motors. The planar features of the pump and its fabrication process using soft lithography make it suitable for integrating into lab-on-a-chip applications.

## 2. Design and fabrication

**Figure 1.**
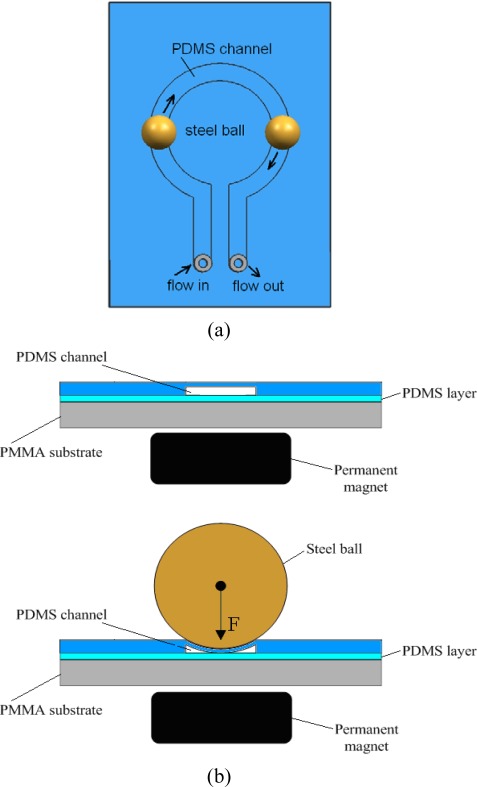
Schematic of the peristaltic micro pump (a) top view; and (b) cross sectional view of the channel before and after placing the steel ball in the presence of magnetic field.

Our peristaltic micro pump consists of a pump chip and a driving setup. The pump chip is composed of a PDMS layer with a circular arc channel bonded on a PMMA substrate. On the top of the PDMS channel steel balls are set. Below the PMMA substrate with a gap, the permanent magnets are placed in proportional spacing, driven by a rotating motor. The number of magnets is the same as that of steel balls. Figure 1 shows a top view and a cross sectional view of the micro pump. The steel balls on the PDMS channel are attracted by the magnetic force and move following the magnets. The PDMS channel with a membrane is staved and peristaltically deformed by the circular movement of the balls, and the peristaltic deformation of the membrane pushes the fluid in the channel, moving it from the inlet to the outlet. By changing the direction of rotation of the DC motor, the fluid can also be pumped backwards in a bidirectional pumping mode.

**Figure 2.**
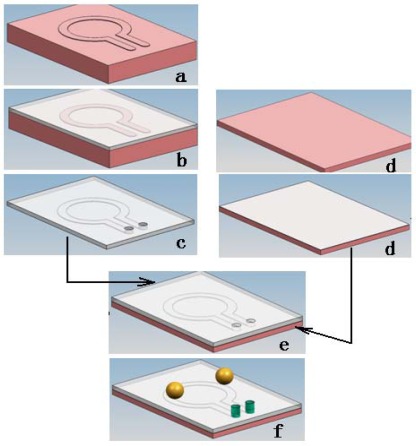
Flowchart of fabrication process, using the PDMS soft lithography technique.

**Figure 3.**
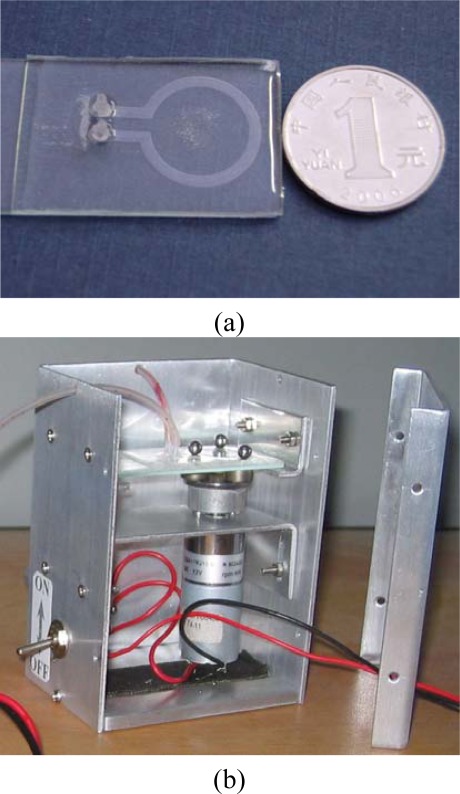
Pictures of a produced micro pump (a) view of a pump chip; and (b) a simple driving setup with a pump chip.

We fabricated a pump chip by standard soft lithography [23] as shown in Figure 2: a) a channel mold was fabricated using CNC milling, with 2 mm width, 300 μm depth, and 10 mm inner diameter, and then two vertical tips were put onto the mold to help forming the inlet/outlet holes later; b) a PDMS precursor (Silgard 184 elastomer, Dow Corning) was mixed with its curing agent at a ratio of 10:1 by weight. After stirring and degassing under 10 Pa vacuum for about 15 min, the PDMS mixture was poured into the mold to form a channel layer; c) after that, the PDMS channel layer was cured at 75°C for 40 min and peeled off from the mold, then the vertical tips were pulled out of the PDMS channel structure, and the inlet/outlet holes were formed; d) q thin PDMS layer of about 20 μm thickness was spin-coated on a PMMA substrate and softly cured at 75°C for 5 minutes [24]; e) the PDMS channel layer was then bonded to the PMMA substrate with a thin PDMS layer and finally the bonded structure was cured at 75°C for 1h; f) silicone tubes with connected tips were fixed into the inlet and outlet; g) a DC motor (12 V, 50 rpm, Ailishi Electronics Technology Center, Beijing, P.R. China), permanent magnets (Nd_2_Fe_14_B, Shenzhen Huapeng Industrial Technology Co., Ltd, P.R. China) and steel balls were also assembled in the pump chip. Figure 3 shows pictures of a produced micro pump chip and the driving setup.

## 3. Results and Discussion

**Figure 4.**
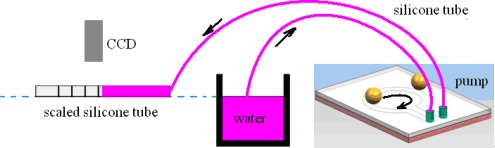
Schematic of the setup for flow rate testing.

Figure 4 shows the experimental setup for flow rate testing. For the pump itself, neodymium magnets (diameter 8 mm, height 4 mm) and steel balls with different diameters from 3 mm to 5 mm were used. The gap between the magnets and the 1.1 mm thick PMMA substrate was about 100 μm. Here the distance between balls and magnets were set to be as small as possible, since the magnetic force will decrease significantly with increasing distance. The whole PDMS structural layer was 450 μm thick. The width, depth and outer diameter of the circular channel were 2 mm, 300 μm and 14 mm, respectively, with inlet/outlet spacing 2 mm.

**Figure 5.**
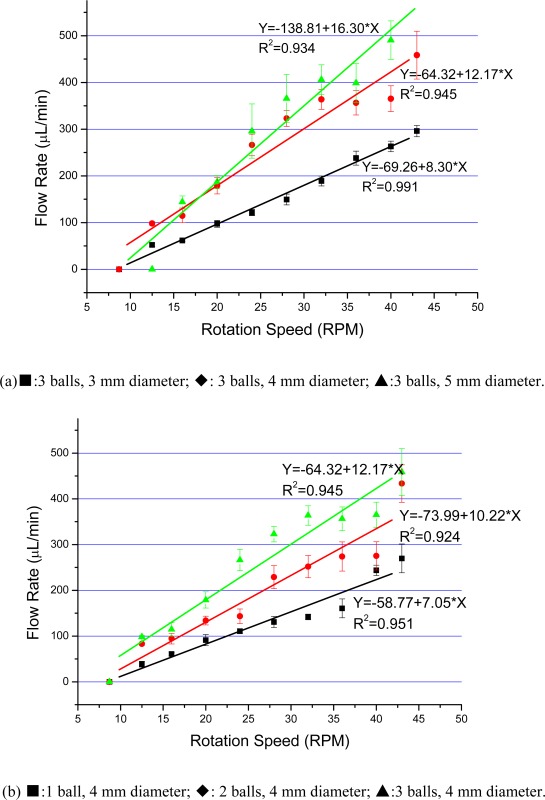
Relationships between flow rate and rotation speed with different (a) ball bearing size; and (b) number of balls at zero back pressure.

For the experimental setup, we used a horizontal scaled silicone tube connected to the outlet and a CCD camera to measure the flow rate. The water level of the inlet container and the outlet tube were kept in a same horizontal level to guarantee zero back pressure. The rotation speed of the DC motor could be adjusted from 0 to 43 rpm by adjusting the supplied voltage from 0 to about 12 V. By changing the rotation speed of the DC motor, the flow rate could be easily adjusted, ranging to about 490 μL/min. Figures 5(a) and (b) indicate flow rates with different ball diameters, and different ball numbers, respectively. In Figure 5 the error bars show the dispersion of 3∼5 measurements, and the lines are obtained by fitting data linearly. It was shown that flow rates increased with higher rotation speed, larger balls and number of balls. It was observed that the pump did not pump out water until reaching a certain rotation speed, since the pump force at a lower rotation speed could not provide enough pressure to conquer the surface tension of the hydrophobic outlet tube. Through primary experiments, we observed that the flow rates decreased at a given rotation speed with increasing backpressure, but the relation between the flow rate and the rotation speed still showed good linearity.

**Figure 6.**
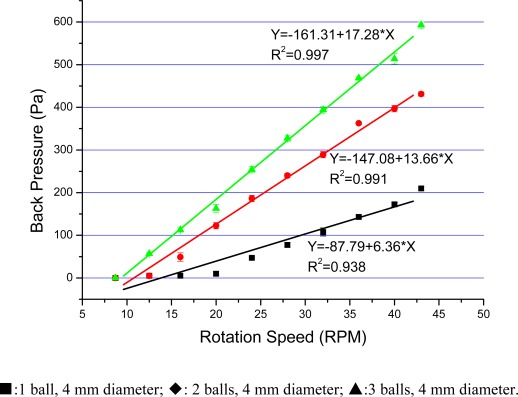
Back pressure vs rotation speed with different ball bearing size.

We measured the back pressure of the pump using a vertical tube. The value of the back pressure was determined by the difference in height of the water columns between the inlet and outlet. As shown in Figure 6, the back pressure increased considerably with higher rotation speed and number of balls, and a back pressure up to 592 Pa was achieved at 43 rpm with three balls of 5 mm diameter. As expected, the back pressure also followed an approximate linear relationship with the rotation speed. Steel balls with diameter larger than 5 mm or numbering more than three are not recommended in our setup, because magnets could not control well the steel balls which would interfere with each other and cause pump failure.

**Figure 7.**
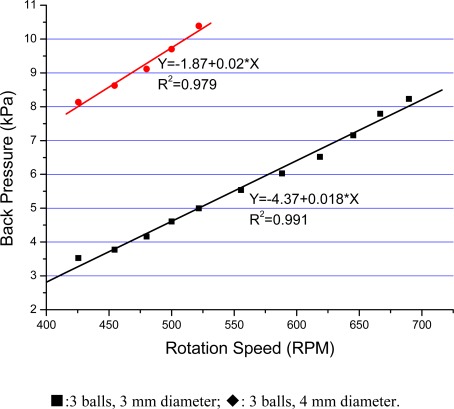
Back pressure vs rotation speed in high speed mode.

In order to demonstrate the possibility of using the pump in high speed mode, we used another DC motor (12 V, 600 rpm, Ailishi Electronics Technology Center, Beijing, P.R. China) with higher speed to replace the primary motor. To our satisfaction, a much higher flow rate up to about 5 mL/min was obtained, and a back pressure was gained up to 10 kPa at around 500 rpm, ten times larger than that using the low speed motor. The relationship of the back pressure and the rotation speed was more linear, compared to low speed mode, as shown in Figure 7. When the rotation speed was higher than 550 rpm, the 4 mm diameter balls could no longer follow the magnets and would fly out. Despite this, the pump could work better and more stably at high speed mode than in low speed mode.

The power consumption of the pump was about 10–180 mW, varying with the operating voltage (1–12 V) with the low speed motor and 50–240 mW with the high speed motor. The pump worked for a total of about 40 hours with no significant performance change being observed. It can be said that the PDMS micro pump can work under stable conditions for much longer lifetime.

**Figure 8.**
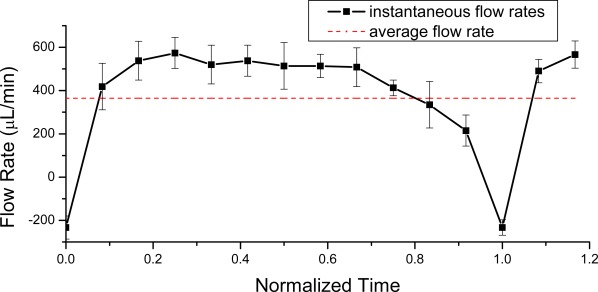
Flow rate during a pumping cycle. The X-axis represents the normalized time of a pumping cycle (time divided by the pumping period), at 40 rpm, 0.5 s pumping period. The data is based on the average of three measurements. The red dashed line represents the average flow rate with the same condition as shown in Figure 5.

To test the smoothness of the pumping flow rate, we measured the dynamic properties of the pump over a single cycle of operation. Flow rates were inferred by tracking the velocity of the colored water head at the outlet tube using a CCD camera with zero back pressure. Figure 8 shows the flow rate during a pumping cycle measured with three 4 mm diameter balls, rotation speed of 40 rpm, and pumping period of 0.5 s (1/3 rotating cycle). Negative flow rates were observed at the end of a period, which indicated back flows. The drop in the flow rates was caused by the periodic jumping of the balls when they moved across the ends of the channel arc.

## 4. Conclusions

Our work demonstrates a simple peristaltic micro pump driven by a rotating motor with magnetically attracted steel balls. The pump produces an adjustable flow rate with relatively smooth pumping. In low speed mode, the flow rate ranged up to 490 μL/min and the back pressure was up to 592 Pa, while in high speed mode, the flow rate can increase to about 5 mL/min and the back pressure achieves values as high as 10 kPa. The two-mode working condition can be better adapted to practical use. Future work will focus on further improving the performance of the pump, especially in a lower flow rate range, reducing the pulsant of the micro pump, and integrating it into a microfluidic chip or lab-on-a-chip.
